# Impactability Modeling for Reducing Medicare Accountable Care Organization Payments and Hospital Events in High-Need High-Cost Patients: Longitudinal Cohort Study

**DOI:** 10.2196/29420

**Published:** 2022-06-13

**Authors:** Maureen A Smith, Menggang Yu, Jared D Huling, Xinyi Wang, Allie DeLonay, Jonathan Jaffery

**Affiliations:** 1 Department of Population Health Sciences University of Wisconsin–Madison Madison, WI United States; 2 Department of Family Medicine and Community Health University of Wisconsin–Madison Madison, WI United States; 3 Division of Biostatistics University of Minnesota Minneapolis, MN United States; 4 University of Wisconsin Health Office of Population Health Madison, WI United States; 5 Department of Medicine University of Wisconsin–Madison Madison, WI United States

**Keywords:** case management, high-risk patients, benefit score

## Abstract

**Background:**

Impactability modeling promises to help solve the nationwide crisis in caring for high-need high-cost patients by matching specific case management programs with patients using a “benefit” or “impactability” score, but there are limitations in tailoring each model to a specific program and population.

**Objective:**

We evaluated the impact on Medicare accountable care organization savings from developing a benefit score for patients enrolled in a historic case management program, prospectively implementing the score, and evaluating the results in a new case management program.

**Methods:**

We conducted a longitudinal cohort study of 76,140 patients in a Medicare accountable care organization with multiple before-and-after measures of the outcome, using linked electronic health records and Medicare claims data from 2012 to 2019. There were 489 patients in the historic case management program, with 1550 matched comparison patients, and 830 patients in the new program, with 2368 matched comparison patients. The historic program targeted high-risk patients and assigned a centrally located registered nurse and social worker to each patient. The new program targeted high- and moderate-risk patients and assigned a nurse physically located in a primary care clinic. Our primary outcomes were any unplanned hospital events (admissions, observation stays, and emergency department visits), count of event-days, and Medicare payments.

**Results:**

In the historic program, as expected, high-benefit patients enrolled in case management had fewer events, fewer event-days, and an average US $1.15 million reduction in Medicare payments per 100 patients over the subsequent year when compared with the findings in matched comparison patients. For the new program, high-benefit high-risk patients enrolled in case management had fewer events, while high-benefit moderate-risk patients enrolled in case management did not differ from matched comparison patients.

**Conclusions:**

Although there was evidence that a benefit score could be extended to a new case management program for similar (ie, high-risk) patients, there was no evidence that it could be extended to a moderate-risk population. Extending a score to a new program and population should include evaluation of program outcomes within key subgroups. With increased attention on value-based care, policy makers and measure developers should consider ways to incorporate impactability modeling into program design and evaluation.

## Introduction

With a national imperative to reduce costs and improve care for high-need high-cost patients [1-3], most accountable care organizations (ACOs) [4] are implementing outpatient case management programs with a nurse or social worker coordinating care. However, there is little evidence of cost savings from these resource-intensive programs [5-7], and they vary widely in design and implementation [5,8]. This widespread implementation of unproven programs has concerned policy makers [9,10] and led to recommendations to design more effective programs [11], to improve the identification of potentially high-cost patients using predictive models [12], and even to abandon care coordination as a cost-saving strategy [13]. For example, case managers often identify patients for enrollment in a case management program using a predictive risk score to find patients at high risk of poor outcomes, such as hospital admission [14].

Rather than attempting to identify effective case management programs and standardize implementation across health systems, a fundamentally different strategy would identify patients who benefit from specific case management programs as they are implemented in practice and match patients to the most beneficial program [5]. Described by Lewis et al as “impactability modeling” [15], this pragmatic approach predicts who is likely to benefit from a particular intervention with respect to an outcome and not who is likely to have a poor outcome. Different from risk scores, these “impactability” or “benefit” scores identify patients who are likely to benefit from enrollment into case management with respect to a specific outcome (eg, preventing hospital admissions). In this way, a benefit score could allow further partitioning of a high-risk population of patients into those who are and those who are not likely to benefit from case management. For example, a high-risk patient may or may not be likely to avoid hospitalizations if enrolled into case management. This additional stratification of a high-risk population using a benefit score may help an ACO target patients for enrollment into case management. Early analyses on impactability in the Medicare population [16] (labeled “benefit score”) and in the Medicaid population [17] (labeled “impactability score”) successfully developed scores to identify individuals who were more likely to benefit from certain case management programs and suggested significant savings [16]. However, neither score was evaluated to determine if it could extend beyond the case management program and population on which it was developed.

The promise of impactability modeling is based on substantial evidence that patients may be more or less likely to benefit from a specific intervention depending on their personal and clinical characteristics [5], although this tailoring of program enrollment may come at a cost. Because impactability models are intrinsically linked to the specific program and population used in their development and because there is wide variation in case management program design and implementation [5,8], it is unclear whether these scores could extend to new programs or populations. To address this question, we evaluated the impact on Medicare ACO savings from a case management “benefit score” developed using a historic case management program enrolling high-risk patients (published elsewhere [16]), and compared the results to prospectively implementing the score in a new case management program enrolling both high- and moderate-risk populations. Our work extends analyses conducted under a Patient-Centered Outcomes Research Institute (PCORI)-funded health systems demonstration grant (HSD-1603-35039; “Variation in case management programs and their effectiveness in managing high-risk patients for Medicare ACOs” [18]).

## Methods

### Study Design and Setting

We used a longitudinal cohort study design with multiple before-and-after measures of the outcome for each case management patient and matched comparison patient [19-21]. Linked electronic health record (EHR) and Medicare enrollment and claims data were extracted from January 1, 2012, through April 30, 2019, to characterize patients during a 1-year baseline period and up to a 1-year follow-up period, as well as census data from the 2007-2011 American Community Survey. The setting was UW Health, a large health system in Wisconsin with 30 statewide academic and community-based primary care clinics and 279 primary care providers. UW Health began participating in the Medicare ACO program in 2013 and, as part of its commitment to become a learning health system [22], began developing scores to support targeted enrollment of patients into population management programs and regularly evaluating program outcomes.

### Ethical Considerations

This project was deemed exempt from institutional review board oversight at the University of Wisconsin–Madison as it constitutes quality improvement or program evaluation [23]. Institutional review board review was not required because, in accordance with federal regulations, the project does not constitute research.

### Case Management Patients

**Figure 1:**
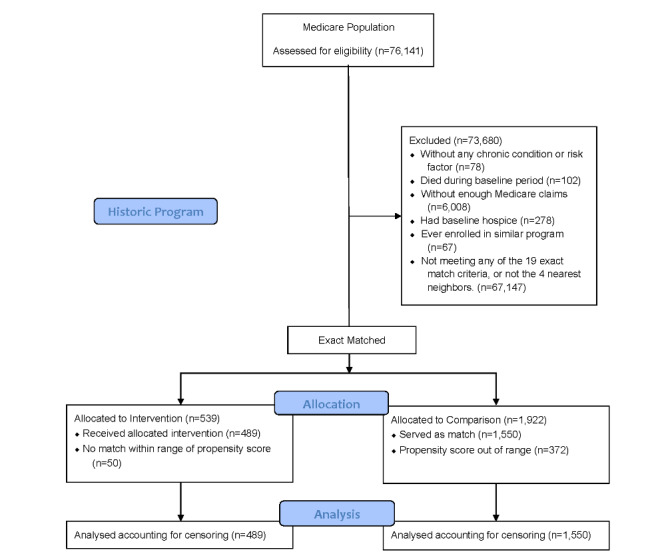
Final analysis sample for the historic case management program.

**Figure 2:**
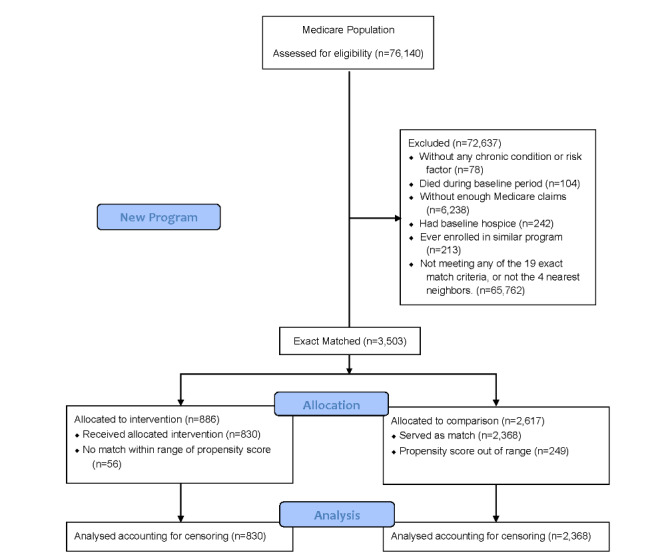
Final analysis sample for the new case management program.

For ease of comparison, we described patients from both the historic [16] and new case management programs. We included patients aged 18 years or older enrolled for at least 1 month with (1) continuous EHR and claims data available for at least 1 year prior to enrollment in case management; (2) assignment to the ACO during baseline and follow-up periods; and (3) at least 1 month of continuous EHR and claims data during the follow-up period. Patients were excluded if they were enrolled in hospice, were on dialysis, or had end-stage renal disease during baseline. Patients were recruited for the historic case management program from June 2013 to December 2018. For the new program, we also excluded any patients previously enrolled in the historic program. Patients were recruited for the new case management program from April 2018 through March 2019. The final sample size of patients in the historic program was 489 (Figure 1) and in the new program was 830 (Figure 2).

### Matched Comparison Patients

We identified all possible comparison patients receiving usual care from the Medicare ACO, who had not been enrolled in the program but who had comparable patient characteristics, had data available, and met the inclusion and exclusion criteria. For each possible comparison patient, we constructed multiple baseline 1-year time periods (63,047 potential comparison patients; 732,799 potential comparison patient-episodes). We matched each case to a maximum of four of the closest eligible comparison patient-episodes. The date at which a possible comparison patient-episode had the closest match to a case with respect to baseline characteristics was the “match date” and was treated identically to the case’s “enrollment date.” The final sample size of matched comparison patient-episodes was 1550 for the historic program (Figure 1) and 2368 for the new program (Figure 2).

### Potential Input Variables

Our strategy leveraged the high-dimensional nature of combined EHR and claims data [24]. For the baseline year for each patient, we constructed 18,406 possible input variables that encompassed sociodemographics (eg, demographics and homelessness), chronic conditions (eg, diagnoses), utilization (eg, procedures and hospitalizations), vital signs (eg, blood pressure), behaviors (eg, tobacco), laboratory values, payments, medications, patient engagement (eg, “no show” appointments), and other information (eg, advance directives). For missing information for continuous variables, we used simple mean imputation within each decile of a hierarchical condition category (HCC) score [25], and for categorical variables, a missing category was created [26]. Continuous variables were transformed into indicators representing “high” and “low” values using the median from the cases. For our core set of descriptive characteristics presented in tables, we used claims data unless otherwise specified. Baseline sociodemographic variables included age (continuous), sex (female/male), race/ethnicity (White/non-White or Hispanic), Medicaid (yes/no), disability entitlement (yes/no), residence (urban, suburban, large town, and small town/rural; categorizing ZIP code from claims) [27], and mean percentage with a high school degree in the 2007-2011 census tract (after geocoding the address from the EHR). Other variables included the HCC score (continuous) [25] and a risk score based on both claims and EHR data that predicted the risk of hospital admission or death within the next 6 months (categorized as “high” risk if the risk was >13%; otherwise, designated as “moderate”) [28]. Chronic conditions included 17 medical conditions defined by Elixhauser et al using International Classification of Diseases, Ninth Revision, Clinical Modification (ICD-9-CM) diagnostic codes, along with an indicator variable for ≥3 of these conditions [29]. Utilization included counts of emergency department (ED) visits, unplanned hospitalizations and hospital days [30], observation stays and observation days, and total Medicare payments. ED visits that resulted in hospitalization were not counted as ED visits but were counted as part of the hospitalization.

### Exact and Propensity Score Matching

To conduct matching, we constructed a high-dimensional propensity score for case management enrollment by adapting the approach from Schneeweiss et al [24]. This included (1) requiring the variables to have a prevalence between 5% and 95% among the cases and a maximum correlation of 0.8 for each covariate (14,909 variables remained); (2) prioritizing covariates using a measure of confounding bias (threshold=95% significance level; 1905 variables remained); (3) selecting covariates using logistic regression with a lasso penalty, with tuning parameters selected using a variant of the traditional stepwise selection, where the final model was chosen on the basis of the best Schwarz Bayesian criterion (37 variables remained) [31]; (4) estimating the propensity score using logistic regression and the 37 predictors, including chronic conditions, HCC scores, procedures, medication counts, telephone encounter counts, etc, for each patient-episode; (5) selecting up to four of the closest eligible comparison patient-episodes using 5 rounds of exact matching (19 exact match variables) and within exact match strata; and (6) selecting final matches using global optimal propensity score matching to minimize the overall distance between propensity scores, using a matrix of distances between all cases and potential matches [32,33]. The quality of our matching process was determined by examining standardized mean differences, which describe a variance-normalized difference in the means of confounders of the control group and the group enrolled in case management. Standardized mean differences with values around 20%-25% were considered moderately imbalanced, but with a range that was amenable to further adjustment through regression [33,34]. Of 1905 baseline variables, 4 had standardized differences between cases and comparison patients above 25%, including the count of unique prescription medication, nonthrombotic nonathlerosclerotic vascular disease or hypertensive heart disease, and professional service payment, and were included in regressions to adjust for residual confounding [33].

### Outcome Measures

Our outcome measures were (1) any unplanned hospital events (admissions, observation stays, and ED visits) during a month, (2) the count of days during the month with any unplanned hospital events, and (3) total Medicare payments during a month, excluding payments for planned hospitalizations and pharmacy payments. We created a data set with 1 observation per patient-episode per month. The first month was 12 months prior to the enrollment/match date and continued for 1 to 12 months after the enrollment/match date until death or censoring due to lack of data.

### Benefit Score

The benefit score [16] differs from a typical risk score in that it predicts the effectiveness or “benefit” of a treatment with respect to the outcome using patient and clinical characteristics (eg, the effectiveness of case management with respect to reducing payments), rather than predicting the outcome directly (eg, payments). This modeling approach was developed under a PCORI methodology grant (ME-1409-21219; “Developing new methods for determining treatment benefits based on individual patient traits” [35,36]). The benefit score represents the estimated reduction in Medicare payments within 1 year if the patient is enrolled in case management [16]. Important variables that determined the benefit from case management included chronic conditions (liver disease, dementia, cardiac dysrhythmias, psychiatric disease, and back disease), count of medication, count of appointment “no-shows,” and use of the electronic medical record patient portal. Patients with negative savings have “no benefit” from case management, and those with positive savings have “benefit.” To provide a qualitative summary of the benefit score, we divided the score into quintiles above 0 (1 to 5) and below zero (−5 to −1). As values close to 0 were ambiguous, scores from 2 to 5 were designated “high benefit” and scores from −5 to 1 were designated “no/low benefit.”

### Statistical Analysis

To estimate the effect of our intervention, our statistical analysis used longitudinal regression modeling of the risk-adjusted difference in outcome trajectories between the cases and comparison patients, using patient-month data. We used an intent-to-treat approach in which individuals who disenrolled from the program were treated as enrolled. We controlled for confounding using exact and propensity score matching (see “Exact and Propensity Score Matching”). After matching, our regression modeling accounted for residual confounding using inverse weighting by the propensity score and for differences in the number of matched comparison patients for each case (ranging from 1-4) by weighting using the inverse of the number of matches. We used the following link functions: logit/binomial (any events), log/zero inflated Poisson (count of event-days), and log/zero inflated gamma (payments). Models included terms for the preintervention trend, change in level, and postintervention trend in monthly events for both cases and comparison patients, and were risk-adjusted for 4 indicator variables with standardized differences above 25% (see above). We stratified our regression analyses of the new case management program by high versus moderate risk, and the final model included benefit category as an interaction term. Treatment of missing data is described in the section on input variables. Results were transformed into predicted outcomes (ie, dollar amount of Medicare payment reduction, number of event-months prevented, or number of event-days prevented) for 100 patients enrolled in case management programs for 1 year, who were similar to those included in our analyses [37]. Because the benefit score was developed on 69% of the cases in the historic program (339 patients enrolled prior to December 1, 2016) [16], intervention effect estimates for the historic program may be biased. We debiased the intervention effect estimation for the historic program using a Harrell bootstrap bias-correction procedure [38], but found no difference after correction and thus presented uncorrected estimates; this procedure is not needed for the new program. We calculated bootstrapped 95% CIs using 400 replications for all outcome models.

## Results

### Characterizing Case Management Programs

The historic case management program used a team approach with a centrally located registered nurse and social worker assigned to each patient and enrolled mostly high-risk patients (Table 1). At program initiation in 2013, patients were identified for further screening using a risk score [28] (calculated monthly) that represented risk of hospital admission or death within 6 months (with “high risk” defined as >13%) or through referral by their primary care provider. After initial identification, patients were screened by nurses or social workers using an assessment tool [39]. Beginning in 2017, the benefit score was also used to identify patients for further screening (with high benefit defined as greater than US $1200 estimated reduction in Medicare payments) [16,40].

The new program relied on nurses physically located in each primary care clinic. Social workers were available only through referral and, in practice, consulted infrequently. The program enrolled both high- and moderate-risk patients. At program initiation, the health system decided to identify 80% of patients through the monthly benefit and risk scoring process developed for the historic program and 20% through the primary care provider referral process [22].

**Table 1:** Case management program characteristics.

Characteristic		Historic program	New program
Established (year)		2013	2018
Enrolled patients, n		550	3600
**Case manager**			
	Profession	RN^a^+SW^b^ dyad	RN
	Training	CCMC^c^	In-house
	Number	15	40
**Case finding process**			
	Risk score	Yes	Yes
	Referrals from providers	Yes	Yes (20% of cases)
	Benefit score	Beginning 2017	Yes (80% of cases)
**Intake process**			
	Comprehensive assessment	Within 30 days	Within 30 days
	Collaborative goal-setting	Within 60 days	Within 60 days
**Intervention intensity**			
	Contacts (#)	3 per month	1 per month
	Duration, mean	160 days	200 days
	Caseload	20-25 primary; 40-50 secondary	75 primary
**Care integration**			
	Physician collaboration	Yes	Yes
	Mode of contact	Telephone and in-person	Telephone and in-person
	Program location	Central	Primary care clinic
**Quality assurance**			
	Medical director attends weekly case consults	Yes	No
	Medical director chart review	Yes	Yes

^a^RN: registered nurse.

^b^SW: social worker.

^c^CCMC: Commission for Case Manager Certification.

### Characterizing Case Management and Comparison Patients

After matching and propensity score weighting, case management and comparison patients were similar with respect to a predetermined set of baseline sociodemographic, chronic condition [29], behavioral, and utilization variables, although cases had slightly more anxiety than comparison patients (Table 2). However, patients in the historic and new programs differed. Patients in the new program were older but less likely to live in an urban area, have Medicaid, or have disability entitlement. They also had less alcohol or drug abuse, less depression but more hypertension and diabetes with complications, lower HCC scores, and less baseline utilization, and were less likely to be high risk. Specifically, 70% of cases in the historic program were high risk compared with 58% of cases in the new program, and the median risk score for cases in the historic program was twice as high as that for cases in the new program (32% vs 16%; data not shown).

Because of these differences, we stratified cases in the new program by high risk versus moderate risk (Table 3). High-risk patients in the new program had similar or slightly higher HCC scores compared with the scores of high-risk patients in the historic program, but were older, less likely to be on Medicaid, more suburban, and more likely to have 3 or more chronic conditions. Moderate-risk patients in the new program had lower HCC scores compared with the scores of high-risk patients and were less likely to have chronic conditions but were more likely to have anxiety and depression.

**Table 2:** Sociodemographics, chronic conditions, and baseline utilization for historic and new case management and matched comparison patients.

Characteristic		Historic program^a^		New program^a^	
		Cases (N=489)	Comparisons (N=1550)	Cases (N=830)	Comparisons (N=2368)
**Sociodemographics**					
	Age (years), mean (SD)	67 (20)	68 (13)	76 (11)	76 (12)
	Female, n (%)	320 (65)	987 (64)	542 (65)	1564 (66)
	Non-White or Hispanic, n (%)	66 (13)	121 (8)	42 (5)	128 (5)
	Medicaid insurance ever, n (%)	219 (45)	643 (42)	184 (22)	594 (25)
	Disability entitlement, n (%)	218 (44)	611 (39)	144 (17)	408 (17)
**Rural/urban, n (%)**					
	Urban code	387 (79)	1031 (67)	508 (61)	1609 (68)
	Suburban	56 (12)	284 (18)	180 (22)	425 (18)
	Large town	35 (7)	186 (12)	128 (15)	295 (12)
	Small town/rural	10 (2)	45 (3)	14 (2)	38 (2)
Percentage with a high school degree, mean (SD)		1 (0)	1 (0)	1 (0)	1 (0)
HCC^b^ score, mean (SD)		3 (3)	3 (2)	2 (1)	2 (1)
High risk, n (%)		344 (70)	997 (64)	483 (58)	1341 (57)
**Chronic conditions, n (%)**					
	3 or more chronic conditions	420 (86)	1322 (85)	757 (91)	2045 (86)
	Congestive heart failure	203 (42)	650 (42)	322 (39)	862 (36)
	COPD^c^/asthma	224 (46)	709 (46)	375 (45)	906 (38)
	Chronic kidney disease	220 (45)	728 (47)	346 (42)	964 (41)
	Alcohol or drug abuse	159 (33)	328 (21)	133 (16)	370 (16)
	Anxiety	274 (56)	671 (43)	393 (47)	825 (35)
	Depression	234 (48)	699 (45)	304 (37)	830 (35)
	Diabetes with complications	125 (26)	396 (26)	188 (23)	479 (20)
	Diabetes without complications	40 (8)	138 (9)	84 (10)	246 (10)
	Hypertension	296 (60)	1044 (67)	676 (81)	1642 (69)
	Liver disease	45 (9)	112 (7)	59 (7)	84 (4)
	Fluid/electrolyte disorders	227 (46)	681 (44)	339 (41)	867 (37)
	Metastatic cancer	19 (4)	37 (2)	38 (5)	77 (3)
	Obesity	154 (31)	430 (28)	262 (32)	570 (24)
	Psychosis	143 (29)	424 (27)	179 (22)	436 (18)
	Peripheral vascular disease	115 (23)	396 (26)	234 (28)	667 (28)
	Renal failure	129 (26)	470 (30)	209 (25)	613 (26)
	Solid tumor without metastasis	34 (7)	127 (8)	87 (10)	258 (11)
**Baseline utilization**					
	Number of ED^d^ visits, mean (SD)	2.2 (4.97)	2.09 (2.89)	1.07 (1.43)	0.96 (1.44)
	Number of hospitalizations, mean (SD)	1.17 (2.46)	1.11 (1.20)	0.64 (0.93)	0.54 (0.84)
	Number of days in hospital, mean (SD)	5.55 (14.98)	5.30 (7.07)	2.77 (5.11)	2.33 (4.57)
	Number of observation stays, mean (SD)	0.25 (0.83)	0.25 (0.49)	0.17 (0.42)	0.15 (0.41)
	Number of days in observation stay, mean (SD)	0.29 (1.08)	0.33 (0.74)	0.21 (0.57)	0.18 (0.53)
	Medicare payment^e^, mean (SD)	29.43 (54.14)	30.57 (32.50)	21.05 (25.86)	17.37 (22.62)

^a^Numbers were adjusted for varying case control ratios.

^b^HCC: hierarchical condition category.

^c^COPD: chronic obstructive pulmonary disease.

^d^ED: emergency department.

^e^Per US $1000.

**Table 3:** Sociodemographics, chronic conditions, and baseline utilization for new case management and matched comparison patients, by risk.

Characteristic		New program, high risk^a^		New program, moderate risk^a^	
		Cases (N=483)	Comparisons (N=1315)	Cases (N=347)	Comparisons (N=1053)
**Sociodemographics**					
	Age (years), mean (SD)	79 (11)	78 (11)	71 (11)	75 (12)
	Female, n (%)	308 (64)	827 (63)	234 (67)	727 (69)
	Non-Hispanic White, n (%)	462 (96)	1247 (95)	326 (94)	989 (94)
	Non-White or Hispanic, n (%)	21 (4)	68 (5)	21 (6)	64 (6)
	Medicaid insurance ever, n (%)	127 (26)	350 (27)	57 (16)	241 (23)
	Disability entitlement, n (%)	79 (16)	204 (16)	65 (19)	196 (19)
**Rural/urban, n (%)**					
	Urban code	280 (58)	909 (69)	228 (66)	699 (66)
	Suburban	111 (23)	213 (16)	69 (20)	211 (20)
	Large town	82 (17)	170 (13)	46 (13)	128 (12)
	Small town/rural	10 (2)	22 (2)	4 (1)	15 (1)
Percentage with a high school degree, mean (SD)		1 (0)	1 (0)	1 (0)	1 (0)
HCC^b^ score, mean (SD)		3 (1)	3 (1)	2 (1)	2 (1)
High risk, n (%)		483 (100)	991 (75)	0 (0)	363 (34)
**Chronic conditions**					
	3 or more chronic conditions	472 (98)	1241 (94)	285 (82)	810 (77)
	Congestive heart failure	249 (52)	651 (50)	73 (21)	219 (21)
	COPD^c^/asthma	242 (50)	558 (42)	133 (38)	349 (33)
	Chronic kidney disease	268 (55)	665 (51)	78 (22)	303 (29)
	Alcohol or drug abuse	78 (16)	207 (16)	55 (16)	158 (15)
	Anxiety	213 (44)	422 (32)	180 (52)	394 (37)
	Depression	165 (34)	419 (32)	139 (40)	405 (38)
	Diabetes with complications	134 (28)	337 (26)	54 (16)	146 (14)
	Diabetes without complications	38 (8)	129 (10)	46 (13)	121 (11)
	Hypertension	406 (84)	969 (74)	270 (78)	677 (64)
	Liver disease	34 (7)	55 (4)	25 (7)	27 (3)
	Fluid/electrolyte disorders	277 (57)	582 (44)	62 (18)	289 (27)
	Metastatic cancer	29 (6)	60 (5)	9 (3)	17 (2)
	Obesity	142 (29)	329 (25)	120 (35)	238 (23)
	Psychosis	85 (18)	230 (17)	94 (27)	203 (19)
	Peripheral vascular disease	184 (38)	440 (33)	50 (14)	235 (22)
	Renal failure	166 (34)	431 (33)	43 (12)	188 (18)
	Solid tumor without metastasis	53 (11)	160 (12)	34 (10)	100 (10)
**Baseline utilization**					
	Number of ED^d^ visits, mean (SD)	1.31 (1.60)	1.15 (1.62)	0.73 (1.07)	0.72 (1.11)
	Number of hospitalizations, mean (SD)	0.93 (1.06)	0.82 (0.96)	0.24 (0.50)	0.22 (0.51)
	Number of days in hospital, mean (SD)	4.18 (6.11)	3.65 (5.52)	0.80 (1.99)	0.81 (2.39)
	Number of observation stays, mean (SD)	0.22 (0.46)	0.17 (0.44)	0.10 (0.33)	0.13 (0.36)
	Number of days in observation stay, mean (SD)	0.28 (0.67)	0.21 (0.59)	0.10 (0.35)	0.15 (0.43)
	Medicare Payment^e^, mean (SD)	28.43 (28.39)	24.27 (26.33)	10.78 (17.24)	9.37 (13.26)

^a^Numbers were adjusted for varying case control ratios.

^b^HCC: hierarchical condition category.

^c^COPD: chronic obstructive pulmonary disease.

^d^ED: emergency department.

^e^Per US $1000.

### Characterizing Case Management Patients by Benefit Category

Approximately one-third of the cases in the historic program were identified as high benefit, while in the new program, 43% of high-risk and 37% of moderate-risk cases were identified as high benefit (Table 4). High-benefit patients in the historic program had higher HCC scores and baseline utilization but were less likely to be high risk and had less disability entitlement when compared with the findings for no/low-benefit patients in the historic program. They were also less likely to have chronic conditions, including chronic obstructive pulmonary disease (COPD)/asthma and anxiety, but more likely to have diabetes with complications. Among high-risk patients in the new program, high-benefit patients were more likely to be female and less likely to have congestive heart failure, chronic kidney disease, alcohol or drug abuse, or valvular disease when compared with the findings for no/low-benefit patients. Among moderate-risk patients in the new program, high-benefit patients were slightly more likely to be female and were less likely to have Medicaid or disability entitlement, COPD/asthma, and alcohol or drug abuse, but more likely to have higher HCC scores and 3 or more chronic conditions, including obesity.

**Table 4:** Sociodemographics, chronic conditions, and baseline utilization for historic and new case management patients, by benefit category.

Characteristic		Historic program (cases only)		New program (cases only), high risk		New program (cases only), moderate risk	
		High benefit (N=170)	No/low benefit (N=319)	High benefit (N=209)	No/low benefit (N=274)	High benefit (N=129)	No/low benefit (N=218)
**Sociodemographics**							
	Age (years), mean (SD)	64 (15)	68 (14)	79 (11)	78 (11)	73 (9)	71 (11)
	Female, n (%)	107 (63)	211 (66)	147 (70)	161 (59)	91 (71)	143 (66)
	Non-White or Hispanic, n (%)	23 (14)	39 (12)	10 (5)	11 (4)	10 (8)	11 (5)
	Medicaid insurance ever, n (%)	76 (45)	15 (48)	54 (26)	73 (27)	14 (11)	43 (20)
	Disability entitlement, n (%)	69 (40)	18 (56)	37 (18)	42 (15)	21 (16)	44 (20)
**Rural/urban, n (%)**							
	Urban code	137 (80)	241 (75)	117 (56)	163 (59)	85 (66)	143 (66)
	Suburban	19 (11)	45 (14)	47 (22)	64 (23)	27 (21)	42 (19)
	Large town	11 (6)	27 (9)	43 (21)	39 (14)	17 (13)	29 (13)
	Small town/rural	3 (2)	6 (2)	2 (1)	8 (3)	0 (0)	4 (2)
Percentage with a high school degree, mean (SD)		1 (0)	1 (0)	1 (0)	1 (0)	1 (0)	1 (0)
HCC^a^ score, mean (SD)		3 (2)	3 (2)	3 (1)	3 (2)	2 (1)	1 (1)
High risk, n (%)		117 (69)	256 (80)	209 (100)	274 (100)	0 (0)	0 (0)
**Chronic conditions**							
	3 or more chronic conditions	142 (84)	301 (94)	204 (98)	268 (98)	117 (91)	168 (77)
	Congestive heart failure	74 (44)	141 (44)	102 (49)	147 (54)	29 (22)	44 (20)
	COPD^b^/asthma	73 (43)	184 (58)	106 (51)	136 (50)	40 (31)	93 (43)
	Chronic kidney disease	79 (47)	155 (48)	103 (49)	165 (60)	28 (22)	50 (23)
	Alcohol or drug abuse	55 (33)	115 (36)	27 (13)	51 (19)	8 (6)	47 (22)
	Anxiety	88 (52)	207 (65)	86 (41)	127 (46)	70 (54)	110 (50)
	Depression	79 (46)	168 (53)	71 (34)	94 (34)	50 (39)	89 (41)
	Diabetes with complications	43 (25)	98 (31)	51 (24)	83 (30)	20 (16)	34 (16)
	Diabetes without complications	17 (10)	14 (4)	16 (8)	22 (8)	14 (11)	32 (15)
	Hypertension	104 (61)	198 (62)	174 (83)	232 (85)	106 (82)	164 (75)
	Liver disease	9 (6)	57 (18)	20 (10)	14 (5)	11 (9)	14 (6)
	Fluid/electrolyte disorders	74 (44)	182 (57)	124 (59)	153 (56)	24 (19)	38 (17)
	Metastatic cancer	8 (5)	12 (4)	12 (6)	17 (6)	4 (3)	5 (2)
	Obesity	55 (33)	104 (33)	64 (31)	78 (28)	54 (42)	66 (30)
	Psychosis	46 (27)	108 (34)	37 (18)	48 (18)	29 (22)	65 (30)
	Peripheral vascular disease	39 (23)	88 (28)	74 (35)	110 (40)	17 (13)	33 (15)
	Renal failure	48 (28)	84 (26)	59 (28)	107 (39)	16 (12)	27 (12)
	Solid tumor without metastasis	13 (8)	23 (7)	22 (11)	31 (11)	12 (9)	22 (10)
**Baseline utilization**							
	Number of ED^c^ visits, mean (SD)	3.04 (4.20)	2.08 (3.30)	1.33 (1.61)	1.30 (1.60)	0.73 (1.07)	0.72 (1.07)
	Number of hospitalizations, mean (SD)	1.70 (2.13)	1.10 (1.62)	0.88 (0.90)	0.97 (1.17)	0.39 (0.62)	0.15 (0.39)
	Number of days in hospital, mean (SD)	8.96 (14.77)	4.79 (8.36)	4.14 (5.44)	4.21 (6.58)	1.27 (2.48)	0.53 (1.58)
	Number of observation stays, mean (SD)	0.33 (0.66)	0.25 (0.60)	0.21 (0.45)	0.23 (0.47)	0.15 (0.42)	0.07 (0.26)
	Number of days in observation stay, mean (SD)	0.37 (0.77)	0.29 (0.80)	0.25 (0.62)	0.30 (0.72)	0.16 (0.46)	0.07 (0.26)
	Medicare payment^d^, mean (SD)	44.39 (52.84)	27.20 (33.55)	27.62 (26.12)	29.05 (30.04)	15.66 (24.34)	7.89 (10.09)

^a^HCC: hierarchical condition category.

^b^COPD: chronic obstructive pulmonary disease.

^c^ED: emergency department.

^d^Per US $1000.

### Relationship Between Case Management and Outcomes by Benefit Category

Across all patients, enrollment in the historic case management program was associated with 80 fewer events and 368 fewer event-days per 100 enrolled patients, although there was no difference in Medicare payments (Table 5). Among high-benefit patients, enrollment in the historic program was associated with 117 fewer events, 536 fewer event-days, and US $1,151,063 reduction in Medicare payments over the subsequent year per 100 enrolled patients when compared with the findings for comparison patients. Among no/low-benefit patients, there was no association between enrollment and outcomes.

For the new case management program, among high-risk high-benefit patients, enrollment was associated with 65 fewer events per 100 patients, with no difference in event-days or Medicare payments. Among high-risk no/low-benefit patients, there was no association between enrollment and outcomes. Among moderate-risk patients, there was no association between enrollment and outcomes for either high-benefit or no/low-benefit patients.

**Table 5:** Average adjusted predicted outcomes per 100 patients enrolled in case management for 1 year among historic and new case management patients, by benefit category.

Outcome		Overall		High benefit		No/low benefit	
		Value	95% CI	Value	95% CI	Value	95% CI
**Historic case management program**							
	Reduction in the number of unplanned events	80	18 to 161	117	51 to 202	9	−65 to 97
	Reduction in the number of unplanned event-days	368	32 to 820	536	214 to 962	44	−357 to 522
	Medicare savings (from the outcome model) (US$)	663,742	−204,900 to 1,827,605	1,151,063	368,423 to 2,216,791	−272,124	−1,449,624 to 1,179,157
**New case management program (all patients)**							
	Reduction in the number of unplanned events	0	−43 to 44	17	−35 to 61	−12	−57 to 41
	Reduction in the number of unplanned event-days	49	−136 to 245	155	−58 to 365	−24	−248 to 203
	Medicare savings (US$)	−33,840	−828,888 to 740,516	181,660	−741,324 to 1,059,818	−181,032	−964,032 to 658,798
**New case management** **program (high-risk patients)**							
	Reduction in the number of unplanned events	34	−32 to 102	65	1 to 128	11	−64 to 100
	Reduction in the number of unplanned event-days	132	−158 to 447	288	−29 to 596	13	−333 to 411
	Medicare savings (US$)	350,407	−714,876 to 1,526,562	891,572	−291,096 to 1,976,962	−58,524	−1,193,112 to 1,263,905
**New case management program** **(moderate risk patients)**							
	Reduction in the number of unplanned events	−57	−114 to 0.30	−72	−156 to 0.01	−47	−112 to 8
	Reduction in the number of unplanned event-days	−190	−486 to 27	−191	−527 to 62	−189	−549 to 52
	Medicare savings (US$)	−288,552	−1,447,932 to 860,098	−449,688	−2,248,944 to 1,022,261	−182,856	−1,365,516 to 827,178

## Discussion

In this Medicare ACO, we found that reduction in Medicare payments and unplanned hospital events from case management participation were limited to high-risk high-benefit patients. A benefit score [16] was able to identify patients who would benefit from a new program with respect to reducing events, but only among a high-risk population with average HCC scores similar to the population on which the score was developed. The score was not able to successfully identify moderate-risk patients who might benefit.

There are several possible reasons for our findings on applying a previously developed benefit score prospectively to a new case management program and population. A possible explanation for why the score was able to successfully identify high-risk patients who might benefit from a new (and different) program is that while the historic and new case management programs differed in teams of composition and location, core elements of a program may depend more on what is done and not who does it or where it is done. When both nurses and social workers work in a practice, they tend toward different roles (social workers assess social issues and nurses coordinate hospital transitions) [41], but in solo practice, each may provide all essential elements of case management [42]. Conversely, the benefit score was developed in a high-risk population [16], not in the broader high- and moderate-risk population served by the new program. Even though targeting patients for case management using a risk score alone may be insufficient [5,43], case management programs may still be designed to optimize care for patients at a specific risk level, and enrollment of patients with different risks may mean a mismatch between program goals/activities and patient needs [44]. Our findings suggest that this score may be limited to identifying case management patients who would benefit from a new case management program only among a population similar to that on which the score was developed.

The latest projections from the Congressional Budget Office are that the Medicare trust fund will run out of money in 2024 [45]. This is the closest the fund has ever come to insolvency since Medicare was established in 1965 and demonstrates the urgent need to understand how to best provide access to high-quality care while simultaneously controlling costs. In order for impactability modeling to help solve the nationwide crisis in caring for high-need high-cost patients [15], “benefit” or “impactability” scores will need to extend beyond the programs and populations in which they were developed. This study provides a first step toward assessing the feasibility and limits of this extension. Although we found evidence that a benefit score could extend to a new program and a similar risk population, caution is warranted as programs vary widely [5,8] and evidence of successful extension to one program does not necessarily indicate that the score could be extended to another program. Unlike risk models, impactability models are intrinsically linked to both the population and the specific program used in their development. Measurement of “similarity” (how similar is similar enough?) is an important open question [46]. More research is needed to understand the core elements of case management (to identify similar programs) and to streamline identification of similar populations.

There are several limitations to our study. First, we were limited to evaluating the impact of the pandemic in a single large health system with both academic and community clinics. This health system did participate in Medicare ACO programs, indicating that they had a strong base of primary care patients [47]. Examining different programs within a system may mitigate variability in coding and data across systems [48], but could also complicate extension to another system. Moreover, academic systems often serve a different population than community practices, but this health system had a large number of community-based primary care clinics [47]. Second, unmeasured confounding is a limitation of all observational studies. As is the case with any observational study, it is almost never possible to know the direction and magnitude of such unmeasured confounding. However, given our measurement of repeated outcomes both prepandemic and postpandemic, as well as an extensive matching process and the similarity of our matched populations, it is unlikely that any remaining small differences explain our findings. Third, we only followed outcomes for 1 year after enrollment, which may be too short to realize positive outcomes [49]. This may explain our finding in the moderate-risk population, and similar to another study [50], negative findings at 1 year might turn into positive findings at 2 years. Fourth, the study focused only on mortality and unplanned events such as hospitalizations and ED visits. Although we used validated algorithms, the definition of unplanned events likely represented some unavoidable events that may not be directly under the control of the health system. This may have slightly reduced our ability to estimate the impact of the case management programs.

The use of impactability modeling to match specific case management programs with high-need high-cost patients who might benefit is consistent with the call by Bates et al to make predictions actionable for interventions [12,51]. This approach does not rely on identifying effective case management programs and attempting to standardize their implementation nationwide, a daunting undertaking given the wide variation in programs [5,8], resistance to change within health systems [52], and practical challenges in implementing evidence-based interventions [53]. Yet, impactability modeling brings its own challenges, most importantly the limitation of tailoring each model to a specific case management program and population. Enthusiasm for this approach should be tempered until additional research provides robust strategies to identify case management programs and populations that are sufficiently similar to warrant a score’s application. In the interim, extending a score developed for a specific program and population to a different program and population should be accompanied by ongoing evaluation to confirm its applicability. Over time, policy makers and measure developers should consider impactability modeling when designing new programs and metrics.

## Abbreviations

ACO: accountable care organization
COPD: chronic obstructive pulmonary disease
ED: emergency department
EHR: electronic health record
HCC: hierarchical condition category
PCORI: Patient-Centered Outcomes Research Institute
